# Rehabilitation of Post-Cerebral Venous Thrombosis

**DOI:** 10.7759/cureus.33512

**Published:** 2023-01-08

**Authors:** Nadiah M Almutairi, Abdulaziz A Albuhayjani

**Affiliations:** 1 Physical Medicine & Rehabilitation, King Fahad Specialist Hospital, Qassim, SAU; 2 General Practice, King Fahad Specialist Hospital, Qassim, SAU

**Keywords:** cerebral venous thrombosis cvt, cerebral venous thrombosis (cvt), occupational therapy, occupational therapy program, rehabilitation medicine, physical therapy rehabilitation, speech and swallowing therapy, stroke, physical medicine and rehabilitation

## Abstract

Cerebral venous thrombosis (CVT) is rare and only accounts for 0.5% of all reported stroke cases. CVT includes severe headaches with neurological deficits, but the vague presentation of symptoms necessitates efficient clinical examination and imaging for a proper diagnosis. Here, we present a case of this rare type of stroke. Our patient exhibited continuous headaches, further complicated by other neurological deficits. We documented this case to aid in the diagnosis and rehabilitation management of CVT. We aim to demonstrate to physicians the importance of early rehabilitation in such stroke cases and improve the outcome for patients.

## Introduction

Strokes are the second-leading cause of death worldwide and one of the many factors contributing to disability [1]. Approximately 80% of strokes are due to arterial ischemic cerebral infarction, and 10%-20% are hemorrhagic strokes [2]. Cerebral venous thrombosis (CVT) is rare and accounts for 0.5% of all strokes [3]. The pathophysiology of CVT is that thrombi in the cerebral vein can cause back pressure and compression, resulting in edema and even hemorrhage [3]. CVT was discovered early in the nineteenth century from the postmortem examination of a 45-year-old male with a six-month history of severe headaches, epilepsy, and delirium [4]. Ninety percent of cases clinically present severe headaches associated with seizures, focal neurologic deficits, papilledema, and altered consciousness [3-5].

CVT may lead to movement disorders ranging from 1%-4% of all strokes [6]. There are two types: hyperkinetic (often hemichorea-hemiballism) or hypokinetic (often vascular parkinsonism) [7]. Language disorders are also frequent and include aphasia, alexia, and acalculia. Aphasia is the most common; it’s the loss or impairment of verbal communication because of brain dysfunction. Like abnormal verbal expression, patients have difficulty understanding spoken words, writing and reading repetition, and naming [8].

Post-stroke patients require multidisciplinary care, including physical medicine and rehabilitation, neurology, geriatrics, internal medicine, family medicine, and other specialties as needed. Also, therapists specializing in occupational, physical, and speech therapies are essential [9]. Early rehabilitation is recommended and safe after the patient is stable after an acute stroke [10]. This case report focuses on the importance of physical medicine, rehabilitation, and physical, speech, and occupational therapies in the management plan of a post-stroke patient in these rare cases.

## Case presentation

**Figure 1:**
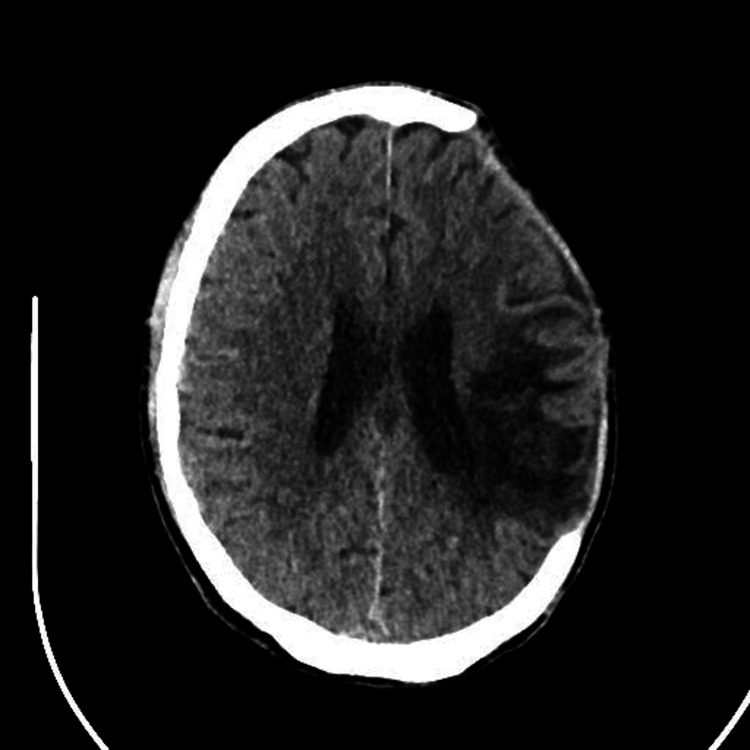
Large hemorrhage in the left parietal lobe, with surrounding edema and effacement of adjacent sulci, as well as a med-line shift to the right.

The patient was a 51-year-old man, a smoker, who was not known to have any chronic diseases. He presented to the emergency room with continuous, severe headaches for three days, right-sided weakness, and slurred speech. Computed tomography (CT) and CT cerebral venography (CTV) showed large hemorrhaging primarily within the left parietal lobe with surrounding edema and effacement of adjacent sulci and the occipital horn of the left lateral ventricle that caused an approximate 3.5 mm med-line shift to the right side (Figure 1).

**Figure 2:**
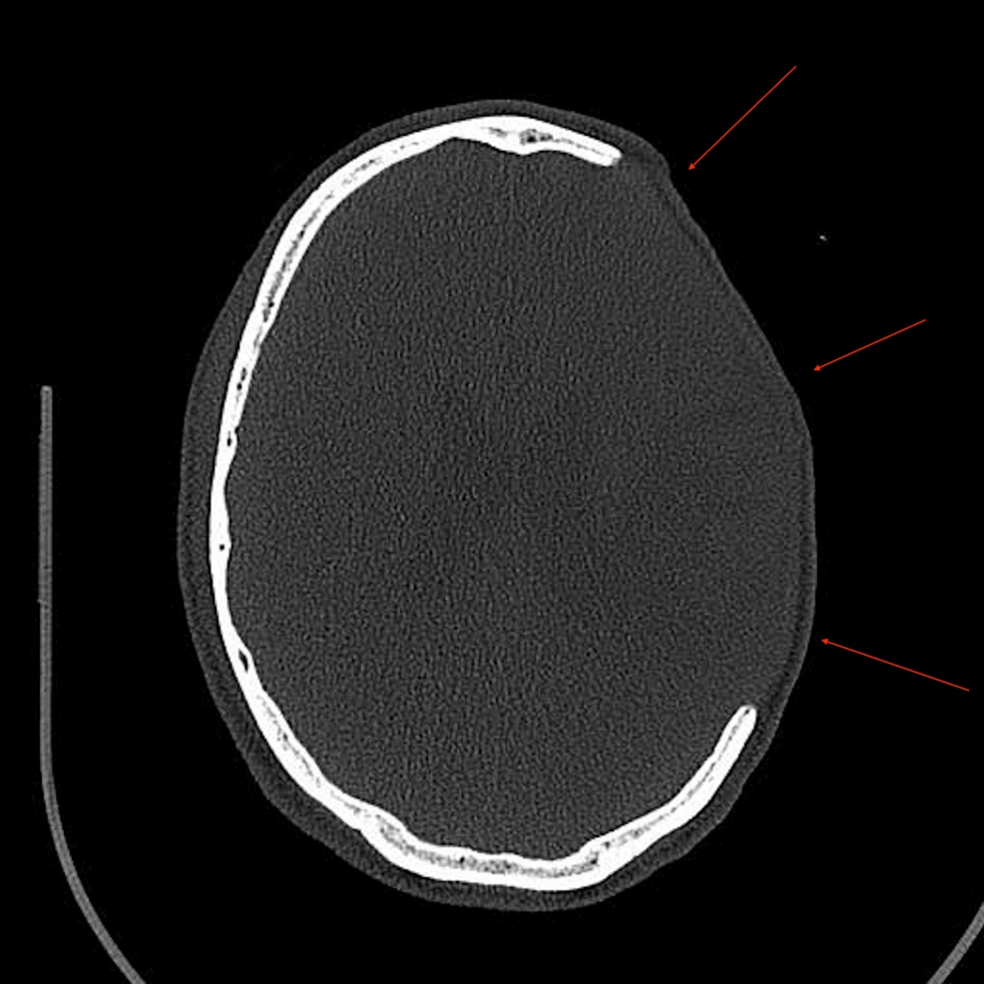
Craniotomy size on the skull.

There was an impending left uncal herniation but no cerebellar tonsillar herniation. Also present was minimal subarachnoid hemorrhaging along the left Sylvian fissure and extensive thrombosis involving the superior sagittal and left-side cerebral sinuses. The patient was admitted to a hospital in the city of Dammam as a case of cerebral venous thrombosis (CVT) with hemorrhagic transformation and uncal herniation, leading to a craniotomy and thrombectomy (Figure 2).

Upon physical examination, the patient was awake, stable, and afebrile, following a one-step command with a Glasgow Coma Scale (GCS) of 14/15. The patient’s difficulty in communicating expressed anomic aphasia. Right-side hemiplegia and hypertonicity were also present. The patient spent two weeks as an inpatient and then came to Qassim to join his family. He was then admitted to our hospital in Qassim for two weeks of extensive rehabilitation therapy. During this time, the patient improved physically as he was trained on sit-to-stand and walking balance, followed by safely walking long distances. He was discharged from physical therapy after achieving the required goals. His upper right still exhibits mild spasticity, which limits the patient's ability to be independent in all activities of daily living. Botulinum toxin injections (Botox) were offered multiple times to relieve the spasticity, but the patient refused treatment.

The patient is currently doing well and is able to use his left hand with good control. Regarding aphasia, the patient has improved from being almost mute to being able to articulate and repeat any given word. His speech has also displayed rapid improvement, especially with his family's support. The patient is fully continent and eating orally without difficulty. There was no history of falling accidents during the patient's admission. The patient lives with family and is well supported. After two weeks, the patient was discharged with good improvement and given an OPD follow-up appointment with a physical medicine and rehabilitation clinic, with physical therapy sessions to continue training and speech therapy sessions for further improvement of language disorders.

## Discussion

Cerebral venous thrombosis (CVT) is rare and accounts for 0.5% of all strokes [3]. It is prevalent in young adults (20-50 years old), and <10% of cases involve individuals older than 65. The clinical presentation of symptoms in CVT is highly variable; a patient can be seen with a one-month history of mild to severe headaches at a clinic or be comatose and admitted to the emergency room. Imaging is required when CVT is one of the potential diagnoses. Magnetic resonance venography and CT venography are both adequate for diagnosis [11].

A Cochrane systematic review of 21 inpatient stroke care trials (3994 participants) demonstrated that multidisciplinary teamwork benefited post-stroke patients. Not only were outcomes improved and mortality reduced, but patients were also more likely to be mostly independent in daily living activities one year after the stroke [12]. Motor impairment is common and has a large impact on a patient's psychological and social aspects. Physical therapy uses exercises and physical activities to condition muscles by restoring strength and movement, help patients with motor disorders, and improve overall life quality by achieving good balance, range of motion, and gait. These physical therapy benefits were evidenced by 30 out of 53 stroke interventions in the Veerbeek et al. study in 2014 [13]. A 2004-2006 study further confirmed these benefits, as 71 patients were able to start early mobilization in less than 24 hours [14].

Occupational therapy helps patients improve, develop, and maintain the needed skills for independence in their daily living and working activities. There is also an increase in the performance of daily living activities and a decrease in poor outcomes like death, deterioration, or dependency [15]. Aphasia is the loss or impairment of verbal communication with a high prevalence in post-acute stroke patients, affecting 21-38% of cases. It nearly always results from left hemisphere lesions, and it is addressed through speech therapy [16]. A meta-analysis was done on 21 studies about the effectiveness of speech and language therapy, finding that patients treated early in the acute period recovered speech skills two times faster than nontreated patients [17].

Spasticity is a stroke complication that results in an increase in muscle tone and a decrease in the ability to use extremities in daily living activities, affecting the patient's gait or hygiene when the hip adductor muscle is spastic. A theoretical study using the Modified Ashworth Scale showed that post-stroke spasticity prevalence was approximately 60% [18]. Spasticity can be treated non-pharmacologically with stretching and physical therapy or pharmacologically with baclofen pills and Botulinum toxin injections, or surgical intervention in severe cases [19]. 

## Conclusions

Cerebral venous thrombosis is a rare type of stroke that can be overlooked or misdiagnosed due to its vague presentation. Proper clinical examination, magnetic resonance venography, and CT venography are necessary to obtain a definitive diagnosis. Physical medicine and rehabilitation play a major role in recovery and maintaining medical stability to prevent strokes from reoccurring. Different stroke types also showed better outcomes if treated and rehabilitated early.
